# General anesthetics protects against cardiac arrest-induced brain injury by inhibiting calcium wave propagation in zebrafish

**DOI:** 10.1186/s13041-017-0323-x

**Published:** 2017-09-04

**Authors:** Dao-jie Xu, Bin Wang, Xuan Zhao, Yi Zheng, Jiu-lin Du, Ying-wei Wang

**Affiliations:** 1 Department of Anesthesiology, Xinhua Hospital, Medical School, Shanghai Jiaotong University, 1665 Kong-Jiang Road, Shanghai, 200092 China; 2 Institute of Neuroscience, State Key Laboratory of Neuroscience, Center for Excellence in Brain Science and Intelligence Technology Chinese Academy of Sciences, Shanghai, 200031 China; 3 Department of Anesthesiology, Huashan Hospital, Fudan University, No. 12 Wu lu mu qi Road, Shanghai, 200040 China

**Keywords:** General anesthetics, Ca^2+^ wave, Cardiac arrest, Brain injury, Zebrafish

## Abstract

Cardiac arrest is a leading cause of death and disability worldwide. Although many victims are initially resuscitated, they often suffer from serious brain injury, even leading to a “persistent vegetative state”. Therefore, it is need to explore therapies which restore and protect brain function after cardiac arrest. In the present study, using Tg (HuC:GCaMP5) zebrafish as a model, we found the zebrafish brain generated a burst of Ca^2+^ wave after cardiac arrest by in vivo time-lapse confocal imaging. The Ca^2+^ wave was firstly initiated at hindbrain and then sequentially propagated to midbrain and telencephalon, the neuron displayed Ca^2+^ overload after Ca^2+^ wave propagation. Consistent with this, our study further demonstrated neuronal apoptosis was increased in cardiac arrest zebrafish by TUNEL staining. The cardiac arrest-induced Ca^2+^ wave propagation can be prevented by general anesthetics such as midazolam or ketamine pretreatment. Moreover, midazolam or ketamine pretreatment dramatically decreased the neuronal apoptosis and improved the survival rate in CA zebrafish. Taken together, these findings provide the first in vivo evidence that general anesthetics pretreatment protects against cardiac arrest-induced brain injury by inhibiting calcium wave propagation in zebrafish.

**Electronic supplementary material:**

The online version of this article (10.1186/s13041-017-0323-x) contains supplementary material, which is available to authorized users.

## Introduction

Cardiac arrest (CA) remains a primary cause of death and persistent disability throughout the world, despite tremendous improvements in emergency medical care and increased public delivery of bystander cardiopulmonary resuscitation (CPR) [1, 2]. Many victims are initially resuscitated, but they often suffer from the extensive post-CA syndrome, including neurologic damage, myocardial dysfunction, and systemic inflammation [3–5]. Among them, brain damage is the greatest proportion of post-CA disability [6], many of CA patients who survive to hospital discharge experience persistent cognitive impairment that profoundly impacts their quality of life [7, 8].

To date, there are no clinically effective pharmacological treatments to protect the brain against ischemic injury. Although therapeutic hypothermia clearly provides a statistically significant improvement of brain damage in ventricular fibrillation–induced out-of-hospital CA patients, the clinical effect is quite modest, it has been shown at most 20% of victims in whom return of spontaneous circulation benefit from hypothermia treatment [9, 10]. Furthermore, the benefit of therapeutic hypothermia on those who experienced nonventricular fibrillation–induced out-of-hospital CA is even less [11, 12]. Therefore, it is essential to decipher the brain injury mechanisms and develop treatments to increase survival and improve quality of life after CA.

Complex cascade reactions are initiated in the brain after CA, including ATP depletion, excessive formation of reactive oxygen, pathological activation of proteases, cell death signaling, inflammation and glutamate-induced excitotoxicity [13, 14]. Different mechanisms contributed at distinct time while contribute to brain injury together. Among them, some mechanisms are executed over hours to days following circulation stops [15, 16]. Most of the current studies have focused on promoting the delayed brain injury recovery after return of spontaneous circulation [17–19]. but there are no fast-acting therapeutic intervention to curtail the extent of brain injury at early stage after CA.

Taking advantage of the larval zebrafish model with the transparency of the brain and heart and the availability of in vivo manipulation [20–22]. We used transgenic Tg (HuC: GCaMP5) zebrafish, in which neuronal activity was indicated by intracellular Ca^2+^ dynamic change, to observe neuronal activity dynamic change during CA by in vivo time-lapse confocal imaging [23]. We first monitored the zebrafish brain generated a burst of Ca^2+^ wave after CA. The Ca^2+^ wave was firstly initiated at hindbrain and then sequentially spread from midbrain to telencephalon. The neurons presented Ca^2+^ overload following Ca^2+^-propagated wave, indicating Ca^2+^-propagated wave caused neuronal death. The CA-induced Ca^2+^ wave can be inhibited by general anesthetics pretreatment. Consistently, general anesthetics pretreatment significantly decreased the neuronal death and improved the survival rate and locomotor activity in CA zebrafish. Taken together, our study reveals the burst Ca^2+^ wave propagation after CA contributed to brain injury. General anesthetics pretreatment can inhibit the CA-induced Ca^2+^ wave and improve neurological outcomes after CA.

## Results

### Cardiac arrest causes a burst of Ca^2+^ wave in zebrafish brain

**Fig. 1:**
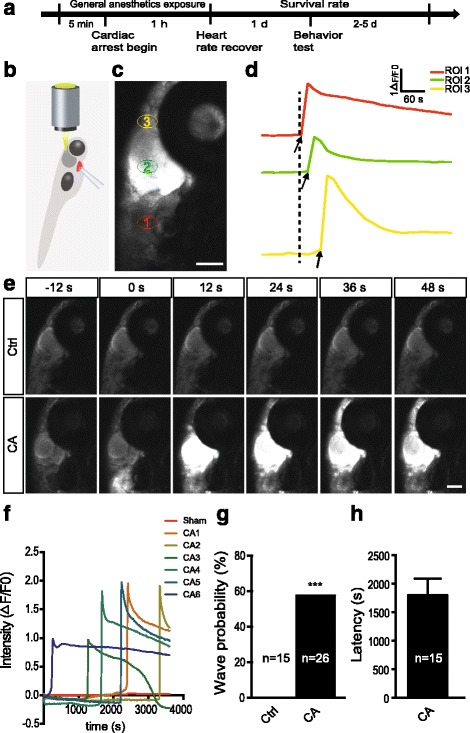
Cardiac arrest causes a burst of calcium wave in brain. **a** Schematic showing the experimental scheme. **b** Schematic showing cardiac arrest (CA) model in zebrafish. A glass micropipette was used to press zebrafish heart (red) until the heart stop beating, neuronal activity was examined with in vivo time-lapse confocal imaging after CA. **c** Calcium activity of Tg (HuC:GCaMP5) transgenic zebrafish in different brain regions after CA (lateral view). ROI1 (red ellipses), ROI2 (green ellipses) and ROI3 (yellow ellipses) marked neuron in hindbrain, midbrain and telencephalon, respectively. Scale bar: 100 μm. **d** Calcium activity of three regions of interest in (**c**) at a higher time resolution. The arrows mark the onset of calcium activities in each region. **e** Time-lapse confocal images showing the lateral view of the brain in live Tg (HuC:GCaMP5) transgenic zebrafish in control and CA zebrafish at 7 day post-fertilization (dpf). 0 s indicating calcium wave onset. Scale bar: 100 μm. **f** The change in fluorescence intensity of neuron in midbrain in individual zebrafish during CA. **g** Occurrence probability of calcium waves in control and CA zebrafish (^***^ *P*<0.001, chi-square test). **h** Quantification of the latency of CA to calcium wave generation in CA zebrafish. Numbers in the histograms represent the number of embryos analyzed in each group

To examine the neuronal activity change in vivo after CA, we used Tg (HuC: GCaMP5) transgenic zebrafish, in which neuronal activity was indicated by intracellular Ca^2+^ dynamic change, to record the real time change of neuronal activity. The zebrafish CA model was established by mechanical pressuring heart until stopping pumping with microelectrode. Neuronal activity was examined with in vivo time-lapse confocal imaging during CA. Nearly all of zebrafish stopped heart pumping within 1 min after pressing (Fig. 1a, b). We found that the zebrafish brain exhibited a burst of Ca^2+^ wave after CA. The Ca^2+^ wave was first initiated in hindbrain, then sequentially propagated to midbrain and telencephalon (Fig. 1c–e; Additional files 1 and 2: Movie S1 and S2). Compared to control zebrafish, 57.7% (15/26) zebrafish brain displayed Ca^2+^ wave after CA (57.7% in CA embryos vs. 0% in control embryos, *P* < 0.001), the latency of CA to Ca^2+^ wave generation was about 1801 ± 291.2 s (Fig. 1f–h). The neuron displayed Ca^2+^ overload following Ca^2+^ wave propagation (Fig. 1f). Taken together, these results suggest that Ca^2+^ wave propagation after CA contributes to neuronal death and hindbrain is likely to more vulnerable to global cerebral ischemia under CA.

### Midazolam or ketamine pretreatment inhibits cardiac arrest-induced Ca^2+^ wave

**Fig. 2:**
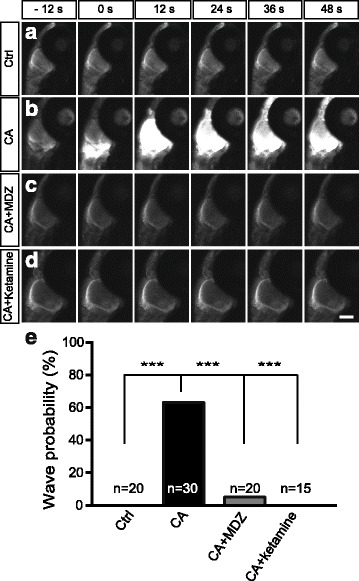
Midazolam or ketamine pretreatment inhibits cardiac arrest-induced calcium wave propagation. **a-d** Time-lapse confocal images showing the lateral view of the brain in live Tg (HuC:GCaMP5) transgenic zebrafish in control (**a**), CA (**b**), CA + midazolam (0.5 mM)(**c**) and CA + ketamine (2.5 mM) pretreatment (**d**) zebrafish at 7 dpf. 0 s indicating calcium wave onset. MDZ, midazolam. Scale bar: 100 μm. **e** Occurrence probability of calcium waves in each group (^***^ *P*<0.001, chi-square test). Numbers in the histograms represent the number of embryos analyzed in each group

Previous studies have been demonstrated that excitotoxic neuronal death occurs in response to cerebral ischemia [24, 25]. To examined whether CA-induced Ca^2+^ wave was associated with the increased neuronal death, we examined whether CA induced Ca^2+^ wave can be prevented by general anesthetics. We pretreated CA zebrafish with midazolam (0.5 mM) or ketamine (2.5 mM) for 5 min before establishing CA model. Compared to CA zebrafish, midazolam or ketamine pretreatment significantly inhibited CA-induced Ca^2+^ wave generation, the occurrence probability of Ca^2+^ waves was 0, 63.3, 5 and 0% in the control, CA, CA plus midazolam and CA plus ketamine zebrafish, respectively (Fig. 2). These results suggest anesthetics can prevent CA-induced Ca^2+^ wave.

### Inhibiting Ca^2+^ wave generation decreases neuronal apoptosis after cardiac arrest

**Fig. 3:**
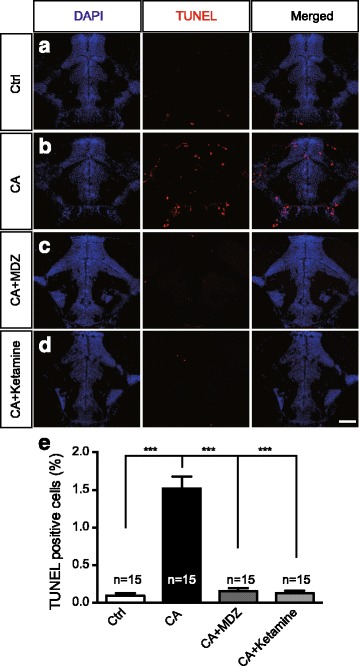
Midazolam or ketamine pretreatment decreases the neuronal apoptosis after cardiac arrest. **a**-**d** TUNEL staining of control (**a**), CA (**b**), CA + midazolam (0.5 mM)(**c**) and CA + ketamine (2.5 mM) pretreatment (**d**) zebrafish at 7 dpf. Red, TUNEL staining, indicating the apoptotic cell; Blue, DAPI-stained nuclei. Scale bar: 10 μm. **e** Quantification of percentages of TUNEL positive cells in each group (mean ± s.e.m.; ^***^ *P*<0.001, one-way ANOVA plus Newman–Keuls multiple comparison test). Numbers in the histograms represent the number of sections analyzed in each group

Neuronal death is the most serious complications for post-CA syndrome. In the present study, we found neuronal death was increased in CA zebrafish compared to control zebrafish by TUNEL staining (Fig. 3), consistent with the previous notion [26, 27]. To further study whether Ca^2+^ wave was responsible for neuronal apoptosis in CA zebrafish, we inhibited the CA-induced Ca^2+^ wave by midazolam or ketamine pretreatment. Our results showed that midazolam (0.5 mM) or ketamine (2.5 mM) pretreatment significantly decreased neuronal apoptosis in CA zebrafish (Fig. 3e). Taken together, these results further indicate that Ca^2+^ wave propagation after CA contributes to neuronal apoptosis, and midazolam or ketamine pretreatment potentially attenuates neuronal apoptosis after CA.

### Inhibiting Ca^2+^ wave improves survival and locomotor function

**Fig. 4:**
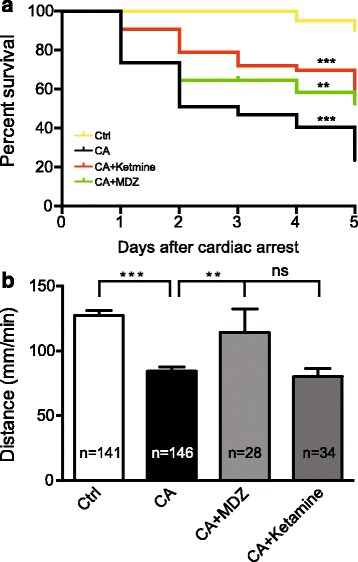
Midazolam or ketamine pretreatment improves survival and locomotor function after cardiac arrest. **a** Survival rate of zebrafish during 5-day follow-up after CA (^**^ *P*<0.01, ^***^ *P*<0.001 vs CA group, log-rank test). **b** Quantification of the spontaneous locomotor distance of 1 day post-CA zebrafish (mean ± s.e.m.; ^**^ *P*<0.01, ^***^ *P*<0.001, ns, no significance, one-way ANOVA plus Newman–Keuls multiple comparison test). Numbers in the histograms represent the number of embryos analyzed in each group

To further determine whether inhibiting CA-induced Ca^2+^ wave can improve the survival and neurologic outcome in CA zebrafish, we first examined survival rate from 1 to 5 day post-CA. The survival rate in CA zebrafish was obviously lower compared to control zebrafish. However, compared to CA zebrafish, midazolam (0.5 mM) or ketamine (2.5 mM) pretreatment significantly increased the survival rate (Fig. 4a).

Locomotor function is a key factor for evaluating neurologic function in zebrafish [28, 29]. To determine whether inhibiting Ca^2+^ wave can improve locomotion of CA zebrafish, we examined spontaneous locomotor function by behavior test. Our results showed the locomotor distance was significantly decreased in CA zebrafish compared to control zebrafish, and midazolam (0.5 mM) pretreatment dramatically increased the locomotor distance (Fig. 4b). However, there was no obviously difference on locomotor distance between ketamine (2.5 mM) pretreatment and CA zebrafish (*P* > 0.05). The spontaneous locomotor distance was 127.3 ± 3.88, 84.3 ± 3.19, 114.3 ± 17.89 and 80.1 ± 6.20 mm/min in the control, CA, CA plus midazolam and CA plus ketamine zebrafish, respectively. Taken together, these results show that general anesthetics pretreatment improves survival rate. Moreover, midazolam pretreatment obviously alleviates neurologic function defect after CA.

## Discussion

In the present study, we provide the first evidence that the brain exhibited burst of Ca^2+^ wave after CA in zebrafish. The Ca^2+^ wave propagation was responsible for brain damage. General anesthetics such as midazolam or ketamine pretreatment can effectively prevent the CA-induced Ca^2+^ wave and neuronal death. Importantly, anesthetics pretreatment obviously improved the neurologic outcome and survival rate after CA. Our study may identify a new therapeutic way to limit on-going brain damage in conditions of CA.

Brain injury is the most complication of post-CA syndromes that severely affects the quality of life [1]. Previous studies have demonstrated that under focal cerebral ischemia (stroke) or traumatic brain injury condition, the ischemic core spread neurotoxicity signal into surrounding undamaged brain regions [30]. The glutamate excitotoxicity has also been extensively studied as a mechanism of spreading toxicity [24, 25, 31]. However, the mechanism of brain damage under global cerebral ischemic in vivo is not clear to state. For example, global cerebral ischemia caused by CA often damages the higher brain but not the brainstem, leading to a ‘persistent vegetative state’ where the patient is awake but not aware [7, 32]. It is suggested that the different brain regions have different vulnerability even though under globally deprived of blood. In the present study, we found that neuronal activity was first increased in hindbrain after CA, then formed a burst of Ca^2+^ wave sequentially propagated to midbrain and telencephalon. The similar mechanism has reported in other organ during death, a recent study demonstrated that the *C. elegans* intestine generated a sequential propagation Ca^2+^ wave during death, inhibiting Ca^2+^ wave propagation can delay stress-induced death [33]. All of these results suggested that the damage signals may be transmited by Ca^2+^ wave propagation during ischemic states. Therefore, these results implied that hindbrain may be vulnerable to ischemic than other brain region in zebrafish under global cerebral ischemia.

Several studies have demonstrated that general anesthetics have neuroprotection under ischemic or trauma brain injury [34–37]. However, the therapeutic window of general anesthetics on neuroprotection after ischemic or trauma brain injury is controversial. To date, most studies focused on neuroprotection of general anesthetics against delayed brain damage post-CA [38, 39]. Research focusing on general anesthetics preconditioning in ischemic brain has been more limited. In the present study, we found pretreatment with general anesthetics ketamine or midazolam before CA effectively decreased neuronal death and improved survival rate in zebrafish after CA, suggesting that general anesthetics preconditioning during CA dramatically improved the neurological outcome.

The mechanism of anesthetics on neuroprotection included attenuation of glutamate excitotoxicity by inhibition of NMDARs or reduction of glutamate release, opening of KATP channels, and activation of neuroprotective signaling pathways and prosurvival gene expression [34, 40, 41]. Based on this and other studies, our results suggest that ketamine (NMDA antagonist) neuroprotection in CA appears to involve limiting NMDA receptor-dependent Ca^2+^ overload. However, the mechanism of midazolam (GABA agonist) on neuroprotection in CA is not clear. Because previous studies have demonstrated that GABA receptors can play a role in neuroprotective action [42]. Furthermore, GABA receptors have significant influence on NMDA receptor activity [43, 44]. Thus, in combination with the results of other studies, it is likely that neuroprotection produced by midazolam pretreatment in CA including actions at both GABAA and NMDA receptor.

In clinical practice, intraoperative cardiac arrest is a catastrophic event that is associated with high mortality [45, 46]. The rescue measures was firstly initiated cardiopulmonary resuscitation (CPR) and corrected the factors of CA [47]. Because most general anesthetics may impair cardiovascular function for causing hypotension and haemodynamic compromise, anesthesiologists usually avoided to administration general anesthetics during CA. In the present study, we demonstrated that early use general anesthetics significantly improved the survival rate by preventing Ca^2+^ wave formation after CA. Therefore, it is interest to perform clinical trials to weight the advantages and disadvantages of early use general anesthetics on neuroprotection and haemodynamic compromise during CA.

CA is a primary cause of death and long term disability worldwide, with patients often unable to return to work, and requiring around the clock care. Brain injury is one of the key factors in determining outcome after CA [1, 7]. Any protective effect against cerebral ischemic injury should improve both survival and neurological outcome of patients after CA. Recently, numerous postresuscitation cerebral protection approaches are currently being investigated experimentally and/or clinically, but most of current studies focused on promoting brain recovery after return of spontaneous circulation [9, 48]. To date, there are no fast-acting therapeutic intervention to reduce mortality and improve the prognosis by curtailing the extent of brain damage within a time frame.

As a vertebrate model system, the zebrafish has emerged as a powerful model for a wide range of human brain disorders [49, 50]. The overall neuroanatomical features and cellular morphology of the zebrafish central nervous system are generally similar to those of mammals [49]. Importantly, the small size and optical transparency of larval zebrafish permits high resolution in vivo time-lapse imaging and manipulation of neuronal activity [51]. Despite many advantages, zebrafish have certain limitations. For instance, some regions in the mammalian brain do not have obvious structural homologous counterparts in zebrafish [52]. Furthermore, it lacks comprehensive behavioral methods to evaluate neurologic function in zebrafish. Therefore, further studies are required to confirm the results in this study in rodents.

In the present study, we report, for the first time, that CA resulted in a burst of Ca^2+^ wave in brain. The Ca^2+^ wave propagation was closely related to neuronal death. General anesthetics pretreatment effectively inhibited Ca^2+^ wave formation, and consequently improved survival rate and neurological outcome of zebrafish after CA. Our study provides a fast-acting therapeutic approach that general anesthetics pretreatment is beneficial to survival rate and neurological outcome after CA. According to above results, it is speculated that early administration general anesthetics during CA contributes to alleviate brain injury. Therefore, it is imperative to carry out the associative clinical study in the future.

## Methods

### Raising and staging Zebrafish embryo

Adult zebrafish (*Danio rerio*) were maintained in the National Zebrafish Resources of China (NZRC, Shanghai, China) with an automatic fish housing system (ESEN, Beijing, China) at 28 °C following standard protocols [20]. Embryos were raised under a 14 h: 10 h light:dark cycle in 10% Hank’s solution, which consisted of (in mM): 140 NaCl, 5.4 KCl, 0.25 Na2HPO4, 0.44 KH2PO4, 1.3 CaCl2, 1.0 MgSO4, and 4.2 NaHCO3 (pH 7.2). Zebrafish handling procedures were approved by Institute of Neuroscience, Shanghai Institutes for Biological Sciences, Chinese Academy of Sciences.

### Cardiac arrest mode of zebrafish

For cardiac arrest mode, Tg (HuC:GCaMP5) transgenic zebrafish were embedded in 1% low melting point agarose in lateral position without anesthetics at 7 day post-fertilization (dpf). A glass micropipette fixed on micromanipulator was used to press zebrafish heart slowly until the heart stop beating. Blood flow in trunk and brain vessel was monitored to evaluate cardiac arrest. Successful CA was accepted when the blood flow ceased in trunk and brain vessel under bright-field microscopy. The cardiac arrest was maintained for about 1 h. To assessing the locomotor activity or survival rate after CA, glass micropipette was removed to allow the recovery of heart beat. Only larvae with successful heart beat were used to subsequent experiment. In control zebrafish, only pericardium, but not heart, were pressed.

### In vivo time-lapse Ca^2+^ imaging

In vivo time-lapse Ca^2+^ imaging experiments were carried out under a 10 objective (numerical aperture (NA), 0.80) with an Olympus FV1000 confocal microscope (Olympus, Japan). Time-lapse images with a resolution of 800 *800 pixels were acquired at 1–2 Hz. All imaging was performed on non-anaesthetized larvae. Before cardiac arrest established, larvae were imaged for 20 min as control, then imaging was lasted about 1 h in each fish after CA. Imaging was performed on zebrafish larvae at room temperature (26–28 °C).

### Analysis of neuronal Ca^2+^ activity

Time-lapse images were processed by ImageJ (NIH), and calcium waves were detected and analysed. Regions of interest (ROIs) of each image were manually marked on the average image calculated from the entire series, and the change in the intensity of each ROI was calculated as (F-F0)/F0, in which F0 was the average intensity of the ROI through control frames. The onset of calcium waves was set if ΔF/F0 in the first frame was >5% of the peak amplitude of the wave during the raising phase [53]. The timing and threshold of each wave were confirmed based on visual inspection for better detection of wave boundaries.

### Drug treatment

Zebrafish was pretreated with midazolam (0.1–0.5 mM) or ketamine (1–2.5 mM) for 5 min before establishing CA model. For keeping effective concentration of drug, the embryo medium was involved the corresponding drug during in vivo time-lapse imaging. For assessing the locomotor function or survival rate, the drug-containing embryo medium was replaced with drug-free embryo medium after in vivo time-lapse imaging.

### TUNEL staining

The brain of larvae was coronally sliced with 100 μm at 7 dpf and TUNEL staining was performed following the manufacturer’s protocol (In Situ Cell Death Detection Kit TMR red, Roche) [54].

### Behavioral testing

All behavioral trials were carried out during the light period in an area at room temperature (28 °C). Behavioral testing consisted of recording larvae in 24-well plates. The video acquisition system used was previously described and implemented for zebrafish behavioral testing [55]. The video acquisition system parameters were set as: Detection threshold: 20,Small/Large movement threshold: 2,“Inact”/Small movement threshold: 0.2. The spontaneous swimming behavior of zebrafish larvae was evaluated at 1 dpf post-CA. Each well of the 24-well plate was filled with 1 ml of embryo water and a single larva was carefully transferred and released in each well center. Following 30 min of acclimation, the locomotor performance was recorded during an hour session and the total distance were measured. Then larvae were maintained as before.

### Statistical analysis

For normally distributed data, statistical analysis was performed using an unpaired two-tailed Student’s *t*-test between two groups and one way ANOVA plus Newman-Keuls multiple comparison test between more than two groups. For non-normally distributed data, Kruskal-Wallis test plus Dunn’s multiple comparison test was performed. For categorical variables, Chi square test was performed. Difference in survival rate was analyzed by the log-rank test. The method used was indicated in each figure legend. Statistical analysis was performed by GraphPad Prism software (GraphPad Software, San Diego, CA) or SPSS version 13.0 (SPSS Inc., Chicago, IL). Summary data are represented as mean ± SD. Differences between groups were considered to be significant at *p* < 0.05.

## Additional files

Additional file 1: The Movie S1. Showing the neuronal activity change in control zebrafish. (MOV 8333 kb)

Additional file 2: The Movie S2. Showing the neuronal activity change in cardiac arrest zebrafish. The zebrafish brain generated a burst of Ca^2+^ wave after cardiac arrest. The Ca^2+^ wave was firstly initiated at hindbrain and then sequentially spread from midbrain to telencephalon. (MOV 8379 kb)

## Abbreviations

CA: Cardiac arrest
CPR: Cardiopulmonary resuscitation
Dpf: Day post-fertilization
MDZ: Midazolam
